# Altered Cytokine Endotoxin Responses in Neonatal Encephalopathy Predict MRI Outcomes

**DOI:** 10.3389/fped.2021.734540

**Published:** 2021-10-12

**Authors:** Mary Isabel O'Dea, Lynne A. Kelly, Ellen McKenna, Tammy Strickland, Tim P. Hurley, John Butler, Claudine Vavasseur, Afif F. EL-Khuffash, Jan Miletin, Lida Fallah, Arthur White, Jason Wyse, Eleanor J. Molloy

**Affiliations:** ^1^National Maternity Hospital, Dublin, Ireland; ^2^Department of Pediatrics, School of Medicine, Trinity College Dublin, Dublin, Ireland; ^3^Trinity Translational Medicine Institute, Trinity College Dublin, Dublin, Ireland; ^4^Department of Paediatrics, Tallaght University Hospital, Dublin, Ireland; ^5^Rotunda Hospital, Dublin, Ireland; ^6^Our Lady's Children's Hospital (CHI), Crumlin, Ireland; ^7^National Children's Research Centre (NCRC), Crumlin, Ireland; ^8^Coombe Women and Infants University Hospital, Dublin, Ireland; ^9^Meso Scale Discovery, Rockville, MD, United States; ^10^Royal College of Surgeons in Ireland, Dublin, Ireland; ^11^Department of Paediatrics, University College Dublin, Dublin, Ireland; ^12^School of Computer Science and Statistics, Faculty of Engineering, Mathematics and Science, Trinity College Dublin, Dublin, Ireland

**Keywords:** neonatal encephalopathy, hypoxic-ischaemic encephalopathy, cytokines, lipopolysaccharide, MRI, neurodevelopment, biomarkers

## Abstract

**Background:** Neonatal encephalopathy (NE) is associated with adverse neurodevelopmental outcome and is linked with systemic inflammation. Pro-inflammatory and anti-inflammatory cytokines are known to play a role in the pathology of NE by activating innate immune cells.

**Methods:** Eighty-seven infants were enrolled including 53 infants with NE of whom 52 received therapeutic hypothermia (TH) and 34 term infant healthy controls (TC). Whole blood sampling was performed in the first 4 days of life, and a 14-spot ELISA Multiplex Cytokine Array was carried out on baseline samples or after stimulation with lipopolysaccharide (LPS) as an additional inflammatory stimulus. The cytokine medians were examined for differences between infants with NE and healthy TC; and then short-term outcomes of Sarnat stage, seizures, and MRI brain were examined within the NE group. The potential of LPS stimulation to predict abnormal MRI was explored using receiver operating characteristic (ROC) curves.

**Results:** At baseline, infants with NE had significantly higher levels of erythropoietin (Epo), interleukin (IL)-6, and IL-1ra and significantly lower vascular endothelial growth factor (VEGF) than had controls. All cytokines were increased after LPS stimulation in infants with NE with an excessive Epo and IL-1ra response than in controls. Infants with NE had lower IL-8, IL-2, IL-6, tumor necrosis factor (TNF)-α, granulocyte-macrophage colony-stimulating factor (GM-CSF), VEGF, and interferon (IFN)-γ than controls had following LPS. GM-CSF and IFN-γ, IL-1β, IL-1ra, and VEGF were higher on days 1–2 in NE infants with abnormal neuroimaging. GM-CSF, IFN-γ, and TNF-α levels with LPS stimulation were different upon stimulation between normal and abnormal neuroimaging. TNF-α is the only strong cytokine predictor both pre- and post-LPS stimulation of abnormal brain imaging.

**Conclusions:** Altered cytokine responses are found in infants with NE vs. controls, and more significant differences are unmasked by the additional stimulus of LPS, which potentially improves the predictive power of these cytokines for the detection of abnormal MRIs. Infants with NE undergoing TH demonstrate both trained immunity and tolerance, and understanding these responses will facilitate adjunctive immunomodulatory treatments.

## Introduction

Neonatal encephalopathy (NE) is associated with neonatal brain injury and has a significant impact on long-term outcome (1). The approximate global incidence of NE is 1.15 million, with 96% of cases accounting for infants with NE born in low- and middle-income countries (2). Infants with NE commonly have multiorgan injury and systemic inflammation in addition to brain injury (3, 4). Cytokines play a key role in the common pathways in NE that are induced by hypoxia–ischemia, reperfusion, metabolic derangements, inflammation, and infection (5). These pathways can cause excessive activation of inflammatory cells of the innate immune response including neutrophils, macrophages, and microglia, which can lead to further inflammation and brain injury (6). Several studies have found differences in cytokine responses in patients with NE, perinatal asphyxia, abnormal neuroimaging, and cerebral palsy (CP), as compared with controls (2, 7–11). Although many studies have investigated the use of biomarkers in the serum of infants with NE to predict brain injury, no single marker was adequately sensitive and specific to diagnose NE or reliably predict outcomes (12). MRI is currently the primary measure of short-term outcome for infants; however, sensitivity is best after 7 days, which is a significant delay for parents and healthcare providers. An ideal biomarker would be measured quickly and in real-time to predict the outcome of infants with NE to dictate the proper course of treatment (13).

The use of lipopolysaccharide (LPS) or endotoxin is a further challenge to the innate immune system and can unmask immune alterations between infants with NE and controls. Understanding the responses of pro- and anti-inflammatory cytokines in NE may facilitate the development of strategies to modulate the inflammatory response and decrease brain injury. We have previously found that in childhood post-NE, there are altered LPS responses and persistent inflammatory cytokine responses and that the cytokine profile correlates to clinical outcome (14, 15). Zareen et al. found increased granulocyte-macrophage colony-stimulating factor (GM-CSF), tumor necrosis factor (TNF)-β, IL-2 IL-6, and IL-8 at school age following NE and an LPS hypo-responsiveness of IL-10, vascular endothelial growth factor (VEGF), erythropoietin (Epo), and TNF-β at school age in children with a history of severe NE (14). Dietrick et al. found higher plasma neurogranin (NRGN), tau, IL-6, IL-8, and IL-10 and lower VEGF and brain-derived neurotrophic factor (BDNF) in NE than in healthy controls, but their cerebrospinal fluid (CSF) biomarker profile did not correlate with clinical outcome (15).

Further research is required in the area of serum biomarkers in term neonates at risk of brain injury to ensure that a sensitive, specific, reliable marker of brain injury is identified to help predict outcome as part of a larger biomarker panel and understand pathogenesis.

We hypothesized that infants with NE have a dysregulated cytokine inflammatory response in comparison with term controls, which would be further elucidated by the addition of LPS *in vitro*. We aimed to investigate the relationship between serial systemic cytokines in infants with NE in comparison with term controls in response to LPS stimulation, and the relationship of their inflammatory profile to short-term outcomes of Sarnat grade, MRI brain result, and seizures.

## Methods

### Ethical Approval

This project involved collaboration and recruitment from the three maternity hospitals in Dublin: the National Maternity Hospital (NMH), Coombe Women and Infants University Hospital (CWIUH), and the Rotunda Hospital. These three hospitals have over 8,000 deliveries per annum and are national centers for therapeutic hypothermia (TH). Ethical approval was received from each institution, and fully informed written consent was obtained from the parents of all infants enrolled. This project was part of the neonatal inflammation and multiorgan dysfunction and brain injury research (NIMBUS), neonatal encephalopathy PhD training network, and neonatal encephalopathy multidisciplinary PhD program (NEPTUNE; www.nbci.ie) (16).

### Study Population

Inclusion criteria of infants with NE undergoing TH and healthy term control infants were enrolled in the study as per our previous publications (2, 17). Neonates at risk of NE, as per Huang et al. criteria (18), were recruited following informed consent and then subsequently divided into Sarnat stage for grade of NE (19). Healthy term control infants undergoing routine phlebotomy on days 1–4 of life were included as controls, but infants undergoing sepsis evaluations or receiving phototherapy for jaundice were excluded.

MRI of the brain was performed on all infants with NE between postnatal days 5 and 12 using a 1.5-Tesla scanner including T1- and T2- weighted imaging, spectroscopy, and diffusion-weighted imaging (DWI). MRI scans were clinically reported independently by pediatric radiologists.

### Sampling and Cytokine Analysis

Whole blood sampling was performed in the first 4 days of life using aseptic technique via central and peripheral arterial lines at times of routine patient phlebotomy. Samples (1–1.4 ml) were collected in sodium citrate tubes and processed immediately. Whole blood was incubated with endotoxin LPS (1 μl/ml for 1 h at 37°C) or phosphate-buffered solution (PBS). Blood was then centrifuged at 1,500 rpm for 10 min at temperature of 4°C. The plasma was then separated and stored at −80°C for later batch processing. The plasma was defrosted and analyzed in two batches for ELISA.

A 14-spot ELISA Multiplex Cytokine Array with Meso Scale Delivery was performed (www.meso-scale.com). Cytokines were analyzed using a multi-spot 96-well. There were five-spot and 10-spot human serum plasma plates customized for the study. The cytokines were examined via a sandwich immunoassay format where capture antibodies were coated in a patterned array on the bottom of the wells of the plate. The plates were then analyzed on the SECTOR Imager and validated. Interferon-gamma (IFN-γ), interleukin (IL)-1β, IL-1α, IL-2, IL-6, IL-8, GM-CSF, TNF-α, TNF-β, IL-18, VEGF, IL-10, IL-1ra, and Epo were studied. The plate was analyzed on an MSD instrument. The results were displayed in pg/ml and analyzed on GraphPad Prism software (https://www.graphpad.com) (7, 20).

### Statistics

Paired comparisons were carried out using Wilcoxon signed-rank tests. Unpaired comparisons were carried out through Wilcoxon Mann–Whitney tests. As this investigation seeks useful prognostic markers of NE outcome, it is necessary to adjust the *p*-values obtained from these tests using a multiple comparison correction (21). Our analysis used the method of Benjamini and Hochberg to correct the *p*-values and adjust for the false discovery rate. A *p*-value was deemed statistically significant if it is less than or equal the significance level of 0.05.

Logistic regression analyses were performed to assess predictive performance. To carry out a receiver operating characteristic (ROC) curve analysis, we first did a log transformation on the data. This makes the data approximately Normal. The range of transformed values is searched for an optimal split, which is used as a threshold to determine the abnormal MRI outcome. Values are then back-transformed to find the thresholds on the original raw scale. The open-source R software was used for statistical analysis.

## Results

### Demographics

Eight-seven infants were prospectively enrolled, including 53 infants with NE (NE-I *n* = 2, NE-II *n* = 40, and NE-III *n* = 11) and 34 healthy neonatal term controls (Table 1). The term control infants had a mean (SD) gestation of 39.2 (1.6) weeks, birth weight of 3.3 (0.5) kg, and Apgar scores of 9 and 10 (±0) at 5 and 10 min; and the group included five male infants (19). There were no statistical differences in gestational age, gender, and birth weights between infants with NE and controls. Fifty-two infants with NE required TH as per total body hypothermia for NE (TOBY) guidelines and were subsequently treated for 72 h (22), and 29 infants had seizures. The NE groups were more likely to have lower Apgar scores than infant controls, with scores lower for NE II/III. MRI scans of the brain were carried out on all the NE groups, and 31 were abnormal.

**Table 1:** Demographics of infants with NE and term controls.

**Variables**		**Control (*n =* 34)**	**NE-I (*n =* 2)**	**NE-II (*n =* 40)**	**NE-III (*n =* 11)**	***p*** **-Value**
GA (week)		39.2 ± 1.6	40.7	39.7 ± 1.7	40.9 ± 2.0	0.21
BW (kg)		3.3 ± 0.5	3.0 ± 0.6	3.3 ± 0.6	3.2 ± 1.2	0.6
Gender, male, n (%)		29	1 (50)	29 (72.5)	5 (45.5)	0.14
Mode of	LSCS	41.2	0 (0)	23 (59)	6 (54.5)	1
Delivery, n (%)	SVD	47	1 (50)	7 (18)	3 (27.3)	0.67
	Inst	11.8	1 (50)	9 (23)	2 (18.2)	1
Apgar @ 1 min		9	3 (2)	2 (8)	1 (6)	0.33
Apgar @ 5 min		10	8 (2)	5 (8)	4 (7)	0.09
Apgar @ 10 min		10	9 (0)	7 (7)	5 (9)	0.02
TH, n (%)		No	1 (50)	40 (100)	11 (100)	1
Seizures, n (%)		No	No	20 (51.3)	9 (81.8)	0.09
MRI—abnormal, n (%)		Not performed	1 (50)	23 (5)	7 (63.6)	1
Cord arterial pH		Not performed	7.0 ± 0.1	7.0 ± 0.2	6.9 ± 0.2	0.21
Cord arterial BE		Not performed	−14.6 ± 2.5	−10.8 ± 6.2	−17.0 ± 6.3	0.06
CPR, yes (%)		No	0 (0)	12 (30.8)	4 (36)	0.73
Intubated, yes (%)		No	0 (0)	25 (64.1)	9 (81.8)	0.3

*Note. The numerical values in results columns represent the mean and standard deviation. p-Value = (95% CI) NE-II vs. III*.

*GA, gestational age; BW, birth weight; LSCS, lower-segment caesarean section; SVD, spontaneous vaginal delivery; Inst, instrumental delivery; TH, therapeutic hypothermia; BE, base excess; CPR, cardiopulmonary resuscitation; NE, neonatal encephalopathy*.

### Cytokine Levels of Neonatal Controls and Neonatal Encephalopathy at Baseline

**Figure 1:**
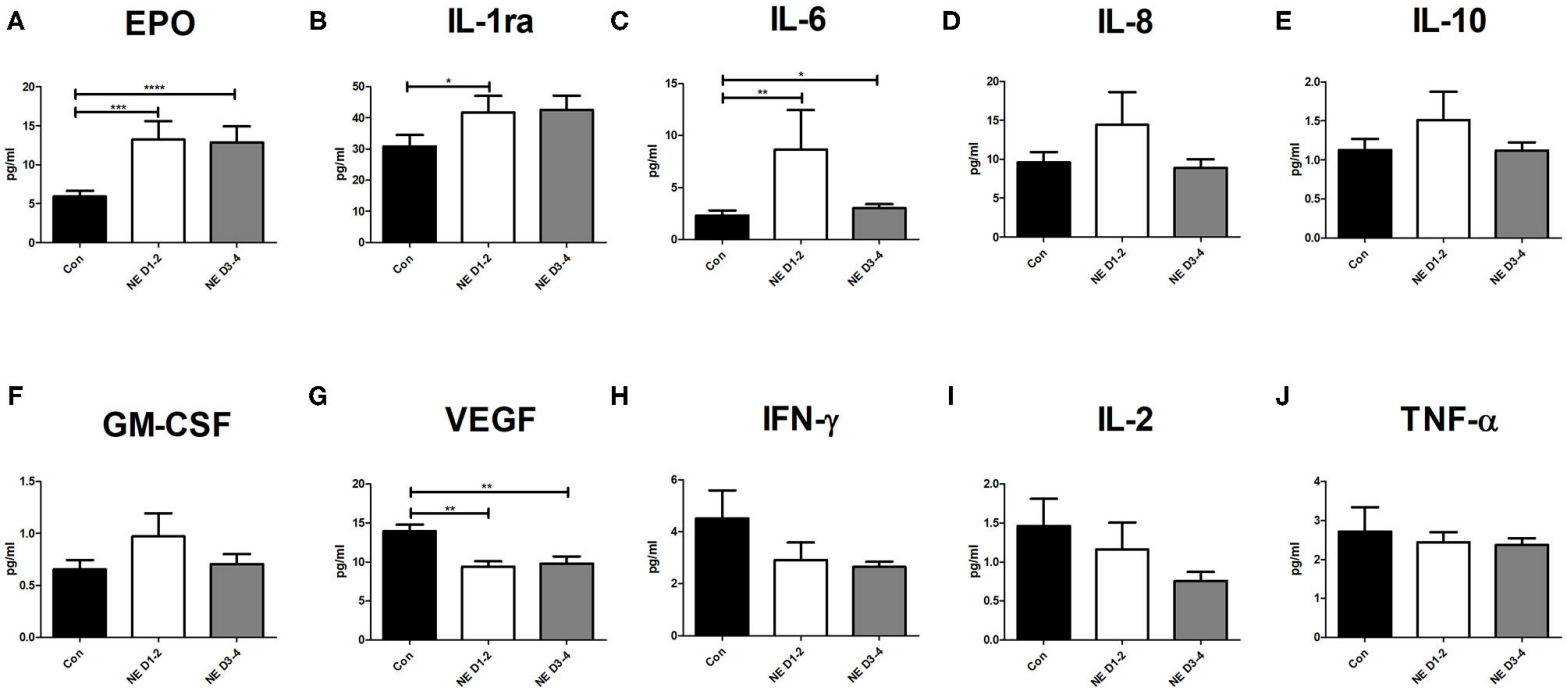
Cytokine levels in NE and control at baseline. Whole blood sampling was performed in the 1st week of life, and a 14-spot ELISA Multiplex Cytokine Array was performed. Graphs represent pg/ml of each cytokine measured in samples of control, NE days 1–2, and NE days 3–4. Cytokines measured are **(A)** Epo, **(B)** IL-1ra, **(C)** IL-6, **(D)** IL-8, **(E)** IL-10, **(F)** GM-CSF, **(G)** VEGF, **(H)** IFN-γ, **(I)** IL-2, and **(J)** TNF-α. p* = *p* < 0.05, p** = *p* < 0.01, p*** = *p* < 0.001, p**** = *p* < 0.0001. NE, neonatal encephalopathy; Epo, erythropoietin; IL, interleukin; GM-CSF, granulocyte-macrophage colony-stimulating factor; VEGF, vascular endothelial growth factor; IFN, interferon; TNF, tumor necrosis factor.

Infants with NE had significantly higher levels of Epo on days 1–2 (*p* = 0.0005) and days 3–4 (*p* < 0.0001) of life than had controls (Figure 1A). Infants with NE showed significantly higher IL-1ra at days 1–2 than did neonatal controls (*p* = 0.04), increasing on days 3–4 (Figure 1B). Infants with NE had significantly higher IL-6 at days 1–2 (*p* = 0.003) and days 3–4 (*p* = 0.03) than neonatal controls (Figure 1C). There were no significant differences between infants with NE and controls for IL-8, IL-10, and GM-CSF (Figures 1D–F). Infants with NE had significantly lower VEGF at days 1–2 (*p* = 0.0003) and days 3–4 (*p* = 0.0003) than neonatal controls; and IFN-γ, IL-2, and TNF-α displayed similar trends (Figures 1G–J).

### Cytokine Levels of Neonatal Controls and Neonatal Encephalopathy Following Lipopolysaccharide Stimulation

**Figure 2:**
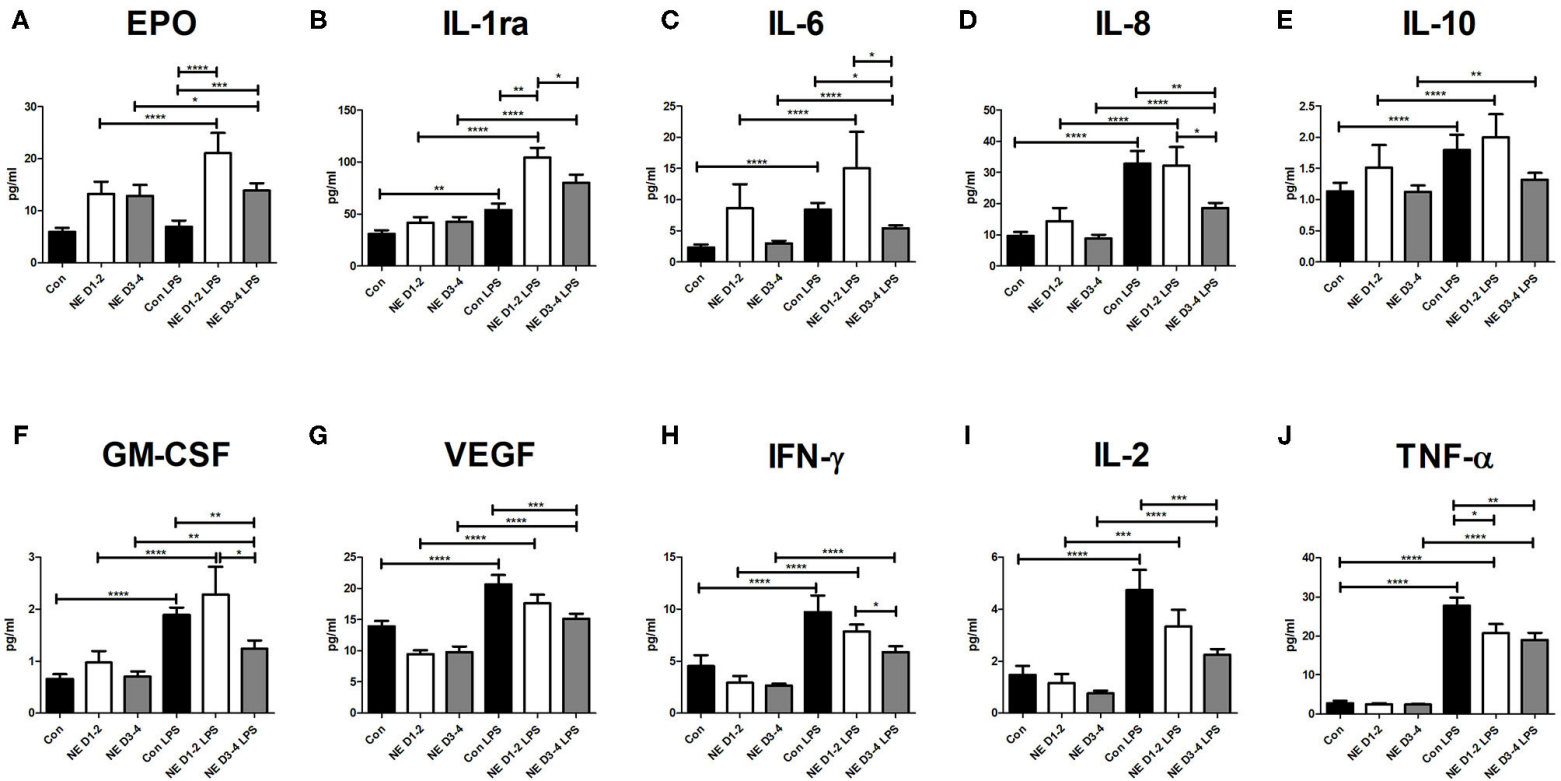
Cytokine levels in NE and control after LPS stimulation. Whole blood sampling was performed in the 1st week of life, samples were stimulated with 1 μg/ml of LPS, and a 14-spot ELISA Multiplex Cytokine Array was performed. Graphs represent pg/ml of each cytokine measured in samples of control, NE days 1–2, and NE days 3–4. Cytokines measured are **(A)** Epo, **(B)** IL-1ra, **(C)** IL-6, **(D)** IL-8, **(E)** IL-10, **(F)** GM-CSF, **(G)** VEGF, **(H)** IFN-γ, **(I)** IL-2, and **(J)** TNF-α. p* = *p* < 0.05, p** = *p* < 0.01, p*** = *p* < 0.001, p**** = *p* < 0.0001. NE, neonatal encephalopathy; LPS, lipopolysaccharide; Epo, erythropoietin; IL, interleukin; GM-CSF, granulocyte-macrophage colony-stimulating factor; VEGF, vascular endothelial growth factor; IFN, interferon; TNF, tumor necrosis factor.

Almost all cytokines increased with LPS stimulation in both term control infants and NE with the exception of Epo, which only increased in infants with NE. Infants with NE on days 1–2 (*p* < 0.0001) and days 3–4 (*p* = 0.02) showed significantly higher Epo after LPS stimulation, but controls did not show any change after LPS (*p* = 0.17; Figure 2A). Cytokines IL-8, TNF-α, IFN-γ, VEGF, and IL-6 were significantly increased after LPS stimulation for the neonatal controls and infants with NE on days 1–2 and days 3–4 (all *p* < 0.0001; Figure 2). IL-1ra was significantly increased after LPS stimulation in the controls (*p* < 0.0001) and infants with NE on days 1–2 (*p* < 0.0001) and days 3–4 (*p* = 0.04; Figure 2B). IL-10 was significantly increased after LPS stimulation in the control (*p* < 0.0001) and in NE on days 1–2 (*p* < 0.0001) and days 3–4 (*p* = 0.007; Figure 2E). GM-CSF was significantly increased after LPS stimulation in the control (*p* < 0.0001) and in NE on days 1–2 (*p* < 0.0001) and days 3–4 (*p* = 0.003; Figure 2F). IL-2 was significantly increased after LPS stimulation in the controls (*p* = 0.0001) and infants with NE on days 1–2 (*p* = 0.0006) and days 3–4 (*p* = 0.0001; Figure 2I).

Infants with NE had lower levels of IL-8, IL-2, IL-6, TNF-α, GM-CSF, IFN-γ, and VEGF than had the infants after LPS stimulation (Figure 2). Infants with NE displayed significantly lower IL-8 (*p* = 0.006), IL-2 (*p* = 0.0009), VEGF (*p* = 0.0003), IL-6 (*p* = 0.03), and GM-CSF (*p* = 0.002) at days 3–4 after LPS stimulation than did controls (Figure 2). Infants with NE displayed significantly lower TNF-α at days 1–2 (*p* = 0.04) and 3–4 (*p* = 0.006) after LPS stimulation than did controls (Figure 2J). There was significantly higher Epo at days 1–2 (*p* = 0.0002) and days 3–4 (*p* = 0.002) in infants with NE compared with controls after LPS stimulation (Figure 2A). At days 1–2, infants with NE had significantly higher IL-1ra (*p* = 0.002) than had control infants after LPS stimulation (Figure 2B). Higher levels of IL-10 and IL-1ra were observed for infants with NE compared with the control infants after LPS stimulation (Figures 2B,E).

All cytokines were decreased in infants with NE at days 3–4 compared with days 1–2 after LPS stimulation, as follows: IL-8 (*p* = 0.02), IL-6 (*p* = 0.02), IFN-γ (*p* = 0.04), IL-1ra (*p* = 0.04), and GM-CSF (*p* = 0.04; Figure 2). Infants with NE had decreased IL-8, IL-2, IL-6, TNF-α, GM-CSF, IFN-γ, and VEGF and increased Epo, IL-10, and IL-1ra.

### Cytokine Correlations to Short-Term Clinical Outcomes in Neonatal Encephalopathy

TNF-α was higher with increasing grade of Sarnat score from NE II to III (*p* = 0.05). Anti-inflammatory IL-10 decreased with severity of NE, and IL-1ra increased with severity. Following stimulation with LPS, IFN-γ decreased with increasing severity of NE, with the lowest IFN-γ being associated with NE III on days 1–2. On days 3–4, TNF-β following LPS stimulation was significantly higher in infants with NE II in comparison with NE III. There were no differences detected in cytokine profile between infants with NE with and without seizures. The cytokine medians were examined for differences between infants with normal and abnormal MRI brain imaging. Baseline GM-CSF and IFN-γ, IL-1β, IL-1ra, and VEGF were higher on days 1–2 in infants with abnormal neuroimaging. GM-CSF, IFN-γ, and TNF-α levels with LPS stimulation were different upon stimulation between normal and abnormal neuroimaging.

To investigate the potential of LPS stimulation for prediction of abnormal MRI, we carried out an exploratory analysis using ROC curves. We assumed a cytokine was a good predictor if it obtained 75% or above in area under the ROC curve (AUC) for classifying normal/abnormal MRI. TNF-α was consistently a good predictor at days 1–2, days 3–4, and pre- and post-LPS stimulation. TNF-α is the only strong predictor on days 1 to 2 pre-stimulation and one of two post-LPS stimulations. On days 3–4 with LPS stimulation, four cytokines (TNF-α, IL-1-α, GM-CSF, and IL-2) were above 75% AUC, especially IL-2 and GM-CSF, at 100% and 87.5%, respectively. The cytokines that responded to LPS stimulation (in terms of being good predictors), with exception of TNF-α, were not good predictors pre-LPS stimulation (Supplementary Figure 1).

## Discussion

Infants with NE had higher levels of Epo, IL-6, IL-1ra, IL-8, IL-10, and GM-CSF at baseline than had the control infants, and lower levels of VEGF, IFN-γ, IL-2, and TNF-α. Infants with NE showed an excessive production of Epo and IL-1ra as compared with controls at both baseline and after LPS stimulation.

LPS was used in this study to examine the inflammatory response of infants with NE compared with control infants. Prior exposure of innate immune cells to endotoxin causes them to become refractory to subsequent endotoxin challenge, termed as “endotoxin tolerance” (23) or “trained immunity” (24). This prior endotoxin exposure inducing LPS tolerance is a form of innate immune memory and may result in reduced response to inflammatory stimulus, hence reducing the inflammatory cytokine output and causing a relative immunosuppression (25), a response that may be beneficial in preventing excessive inflammation (26). In addition, we found an augmented response to LPS for Epo and IL-1ra, indicating a trained immune response, as defined by Netea et al. as functional reprogramming of innate immune cells by insult exposure leading to an altered response upon subsequent threats (24). An altered neutrophil phenotype was found in neonates with sepsis or perinatal asphyxia who were LPS hypo-responsive (27). Following *ex vivo* hypoxia, neonates did not upregulate neutrophil LPS responses in contrast to adults (28). Munoz et al. demonstrated a reduced LPS-induced production of cytokines of IL-1α, IL-1β, IL-6, and TNF-α production in adult patients with sepsis, which was exacerbated in non-survivors (29). We demonstrated a hypo-responsiveness to LPS in NE infants with lower leucocyte activation markers of CD11b and NOX2 as compared with healthy term neonatal controls (17).

Epo was higher in infants with NE than in control infants in this study at baseline, and following LPS stimulation. Similarly, Sweetman et al. demonstrated increased Epo in infants with NE and that higher levels of Epo were associated with abnormal neuroimaging (7). This has also been corroborated in cord blood by Hagag et al., who found higher Epo levels in cord blood of infants with NE and that the Epo level correlated with severity of NE (30). Epo has been extensively studied in NE as a potential therapeutic agent, as it decreases leucocyte infiltration and prevents IL-1β rise post-exogenous administration in animal models of brain injury (31). Currently, the High-Dose Erythropoietin for Asphyxia and Encephalopathy (HEAL) trial (NCT-02811263) is underway exploring whether high-dose Epo reduces the combined outcome of death or neurodevelopmental disability when given in conjunction with TH to newborns with moderate/severe NE. However, the recent Preterm Erythropoietin Neuroprotection Trial PENUT trial demonstrated no difference in outcomes of preterm infants treated with Epo (32).

VEGF is a growth factor activated by hypoxia (33) and was lower in infants with NE than in controls at baseline and in NE on days 3–4 after LPS stimulation. Lower VEGF was previously associated with severe NE and mortality (7). Aly et al. demonstrated increased VEGF in perinatal asphyxia and further elevated in infants who developed NE; however, this study was done with cord blood rather than postnatal neonatal blood (33).

Anti-inflammatory IL-1ra was increased in infants with NE at baseline and higher than in controls in response to LPS stimulation. Therapeutic IL-1 receptor antagonists have demonstrated reduced injury and an acceptable safety profile in adult stroke (34). IL-10, an anti-inflammatory cytokine, was increased following LPS stimulation in both infant controls and those with NE. Higher baseline IL-10 was associated with severity of NE, which previously was found to be elevated in non-survivors of NE by our group (2). Pang et al. found higher IL-10 in NE compared with controls and that increased IL-10 predicted mortality and adverse early childhood outcome (35). IL-10 agonism has also been demonstrated to reduce white matter damage in neonatal rats following maternal sepsis, suggesting a neuroprotective effect (36). IL-10 when co-administered with endotoxin in animal model of brain injury counteracted the acute effects of LPS on cerebral metabolism (37).

GM-CSF is known to stimulate granulocyte, monocyte, and eosinophil production (38). GM-CSF was higher in NE than in control infants; however, on days 3–4 of life, the level of GM-CSF post-LPS stimulation was higher in controls than in NE. Higher GM-CSF levels have been demonstrated to expand myeloid cell expansion, inflammation, and reactive oxygen species leading to CNS injury independent of T cells in mouse injury model (39). Infants with NE and abnormal neuroimaging had significantly elevated GM-CSF at 0–24 h and an association with death in NE in a study pre-TH (2). Savman et al. found no differences between CSF of GM-CSF controls and infants with NE (40). Sweetman et al. found that day 2 GM-CSF predicted abnormal MRI results in NE and Bayley-III (41). Savman et al. found no differences between CSF of GM-CSF controls and infants with NE (40).

Both infants with NE and controls had increased IFN-γ with LPS stimulation. IFN-γ interacts with brain resident cells to induce neurotrophins and provide a peripheral immune response by increasing neutrophil and monocyte function (42). IFN-γ has been demonstrated to produce neuroprotective factors for myelin sheaths and protect against inflammation (43). These results were corroborated by Okazaki et al., who found that the concentration of IFN-γ in perinatal asphyxia was lower than in controls; however, Massaro et al. demonstrated that a higher level of IFN-γ on day 1 was associated with abnormal neuroimaging in NE (44, 45). Seifert et al. inhibited IFN-γ in mouse model of stroke injury and demonstrated reduced neurodegeneration, indicating its possible future immunomodulation potential (46).

Infants with NE had significantly higher IL-6 at baseline than neonatal controls, but IL-6 was hypo-responsive to LPS in infants with NE on days 3–4. Massaro et al. similarly found higher IL-6 at both 24 and 72 h in NE during TH but that the initial IL-6 profile did not correlate with developmental outcome at 1 year (47). Bharathi et al. found higher cord IL-6 in perinatal asphyxia, which correlated with the severity of asphyxia (48) and had a significant negative correlation with developmental score at 6 months. Foster-Barber et al. found that higher levels of IL-6 in the neonatal period during NE differentiated infants with CP at follow-up but had no correlation with NE severity (10). From a therapeutic point of view, IL-6 blockade with tocilizumab may be a potential adjunctive therapy to decrease the downstream cytokine storm. Tocilizumab has been approved by the Food and Drug Administration (FDA) for use in COVID-19 patients, and the RECOVERY trial has reported decreased mortality and need for mechanical ventilation for those hospitalized due to COVID-19 (49).

IL-8, a chemokine involved in neutrophil recruitment, was increased with LPS stimulation in NE and controls. O'Hare et al. found that IL-8 was significantly elevated at birth in infants with severe NE and abnormal neuroimaging, but this was not replicated in our cohort (2). Foster-Barber et al. found that higher levels of IL-8 in the neonatal period in NE infants differentiated infants with CP at follow-up but did not find a correlation between IL-8 and severity of initial grade of NE (10).

Baseline IL-2 levels were similar in NE infants and controls. Both NE and control levels were increased with LPS; however, IL-2 was relatively hypo-responsive to LPS in NE. Bharathi and Okazaki similarly found no differences in cord blood IL-2 levels between NE and controls (44, 48). IL-2 is a potent immunomodulator, activating immune cells such as T cells, T regulatory (Treg) cells, and natural killer (NK) cells (50). IL-2 receptor antagonists are already used therapeutically in anti-inflammatory and anti-rejection treatments, so they may have a future in immunomodulation in NE (51).

Baseline TNF-α levels were similar in controls and NE, and both increased with LPS. Bharathi similarly found no differences in cord blood TNF-α levels between NE and controls (48). TNF-α was relatively hypo-responsive to LPS in NE infants on days 3–4 in comparison with controls. TNF-α was higher with increasing grade of Sarnat score severity. TNF-α consistently was a good biomarker both pre- and post-LPS stimulation to differentiate normal vs. abnormal neuroimaging in the NE group (Supplementary Figure 1). TNF-α is known to impair the blood–brain-barrier permeability after hypoxia, and anti-TNF agents are commonly therapeutically used for autoimmune and inflammatory disorders, such as rheumatoid arthritis and inflammatory bowel disease, so they potentially represent a target for future immunomodulation (52, 53).

All cytokines increased after LPS stimulation in the NE groups. Epo, however, showed an excessive response to LPS as compared with controls, so careful consideration should be taken when considering Epo as a therapeutic agent. Several cytokines displayed hallmarks of a dampened immune response, and despite that they increased after LPS, IL-8, IL-2, IL-6, TNF-α, GM-CSF, IFN-γ, and VEGF were lower for NE than the infant controls. Several previous studies have found differences in cytokine responses between infants with brain injury and control infants. O'Hare et al. showed elevated GM-CSF, IL-8, and IL-10 and lower TNF-α and VEGF in infants with abnormal neuroimaging (2). Jenkins et al. demonstrated higher early IL-6, IL-8, and IL-10 in NE treated with TH at 24 h in comparison with NE treated with normothermia, and downregulation of IL-6, IL-8, and IL-10 at 36 h in infants with better outcomes (8). Numis et al. demonstrated that increased pro-inflammatory cytokines such as IL-6 and TNF-α were associated with childhood epilepsy following NE (9). IL-1, IL-6, and TNF-alpha were increased in children with CP or who were deceased at 1 compared to those with normal neuromotor outcome following NE (10, 11).

The strengths of this study include the large sample size and the use of term infants as age-matched controls and in the examination of LPS responses. This is in contrast to other studies that use cord blood that is relatively LPS tolerant and is not reflective of postnatal immune responses (54). Olin et al. have demonstrated that cord blood is not representative of postnatal immunity from a longitudinal analysis of 100 neonates over the first 3 months of life (54). Since this study explores the immune phenotype, neonatal blood is a much better comparator to use, albeit difficult to obtain and difficult to do complex experiments on the small volumes that can be ethically obtained in comparison with the abundant availability of cord blood in the maternity hospitals. Olin et al. found that cord blood immune cell correlation to neonatal blood demonstrated only six out of 21 measurements correlated to neonatal blood (54). They reported that the cell composition, plasma protein concentration, and cell phenotypes are all different between the cord blood and neonate blood (54). The weaknesses of the study include relatively small patient numbers on subdivision of the NE cohort for short-term outcomes and the limitation of only presenting short-term outcome data.

The exploration and analysis of the inflammatory phenotype in NE and in term controls have shown a distinct inflammatory phenotype and innate immune response in NE. Infants with NE displayed a relative LPS hypo-responsiveness in comparison with the robust response of the term control infants. Understanding this balance of pro- and anti-inflammatory cytokines ultimately helps in understanding the pathophysiology and identifying ways of modulating the harmful effects and potentiating the beneficial effects (55, 56). Although due to a small number of available cases further exploration is recommended, this is an important early step toward the use of LPS stimulated cytokines as predictive biomarkers.

## Data Availability Statement

The raw data supporting the conclusions of this article will be made available by the authors, without undue reservation.

## Ethics Statement

The studies involving human participants were reviewed and approved by Coombe Hospital Ethics Committee, Rotunda Hospital Ethics Committee, National Maternity Hospital Ethics Committee. Written informed consent to participate in this study was provided by the participants' legal guardian/next of kin.

## Author Contributions

MO'D recruited patients, performed laboratory work, data analysis, and wrote the paper. LK, EMc, TH, and TS performed laboratory work, data analysis, and wrote the paper. JB assisted with laboratory work. CV, AEL-K, and JM assisted with Hospital recruitment and reviewed the paper. LF, JW, and AW assisted with statistical and data analysis. EMo supervised the design and execution of the study, performed the final data analyses, and writing of the manuscript. All authors contributed to the article and approved the submitted version.

## Funding

This work was supported by National Childrens' Research Centre (15136/207634), Health Research Board (HRB Grant No: NIMBUS 14025/205305), and National Children's Hospital Foundation (NCH UNICORN Grant No: 15136/207634).

## Conflict of Interest

JB was employed by MSD. The remaining authors declare that the research was conducted in the absence of any commercial or financial relationships that could be construed as a potential conflict of interest.

## Publisher's Note

All claims expressed in this article are solely those of the authors and do not necessarily represent those of their affiliated organizations, or those of the publisher, the editors and the reviewers. Any product that may be evaluated in this article, or claim that may be made by its manufacturer, is not guaranteed or endorsed by the publisher.

## Supplementary Material

Supplementary Figure 1 ROC curves for cytokines TNF-a **(A,B)**, GMCSF **(C,D)**, IL-1a **(E,F)** and IL-2 **(G,H)** showing their respective AUC, i.e., classification accuracy. Left hand side plots show those for cytokines pre-LPS stimulation and right hand side ones are for post-stimulation.
