# Supplementary figures and images for: Altered Cytokine Endotoxin Responses in Neonatal Encephalopathy Predict MRI Outcomes
Source: Front Pediatr. 2021 Oct 12;9:734540. doi: 10.3389/fped.2021.734540 (PMC8547258; doi:10.3389/fped.2021.734540)

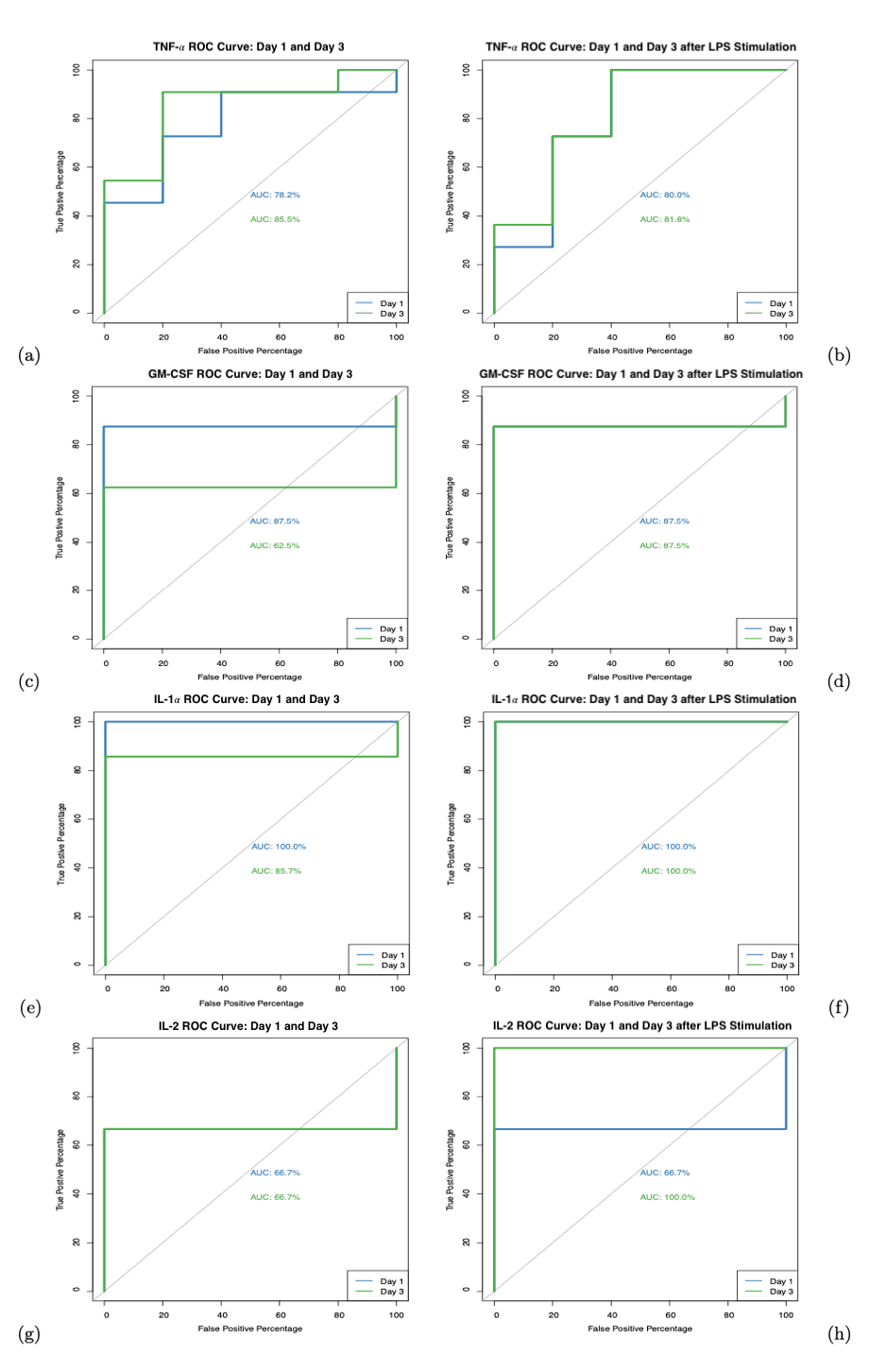

Supplement: Supplementary Figure 1 — ROC curves for cytokines TNF-a (A,B), GMCSF (C,D), IL-1a (E,F) and IL-2 (G,H) showing their respective AUC, i.e., classification accuracy. Left hand side plots show those for cytokines pre-LPS stimulation and right hand side ones are for post-stimulation. [file Image_1.PNG]
